# Selective removal of deletion-bearing mitochondrial DNA in heteroplasmic Drosophila

**DOI:** 10.1038/ncomms13100

**Published:** 2016-11-14

**Authors:** Nikolay P. Kandul, Ting Zhang, Bruce A. Hay, Ming Guo

**Affiliations:** 1 Division of Biology and Biological Engineering, California Institute of Technology, Mail Code 156-29, 1200 E. California blvd., Pasadena, California 91125, USA; 2 Department of Neurology, Brain Research Institute, David Geffen School of Medicine, University of California, Los Angeles, Los Angeles, California 90095, USA; 3 Department of Molecular and Medical Pharmacology, Brain Research Institute, David Geffen School of Medicine, University of California, Los Angeles, Los Angeles, California 90095, USA

## Abstract

Mitochondrial DNA (mtDNA) often exists in a state of heteroplasmy, in which mutant mtDNA co-exists in cells with wild-type mtDNA. High frequencies of pathogenic mtDNA result in maternally inherited diseases; maternally and somatically acquired mutations also accumulate over time and contribute to diseases of ageing. Reducing heteroplasmy is therefore a therapeutic goal and i*n vivo* models in post-mitotic tissues are needed to facilitate these studies. Here we describe a transgene-based model of a heteroplasmic lethal mtDNA deletion (mtDNA^Δ^) in adult *Drosophila* muscle. Stimulation of autophagy, activation of the *PINK1/parkin* pathway or decreased levels of *mitofusin* result in a selective decrease in mtDNA^Δ^. Decreased levels of *mitofusin* and increased levels of ATPIF1, an inhibitor of ATP synthase reversal-dependent mitochondrial repolarization, result in a further decrease in mtDNA^Δ^ levels. These results show that an adult post-mitotic tissue can be cleansed of a deleterious genome, suggesting that therapeutic removal of mutant mtDNA can be achieved.

## Abstract (web summary)

Heteroplasmy, in which mutant and wild-type mitochondrial DNA (mtDNA) coexist in a cell, can result in diseases. Here the authors generate transgenic flies with heteroplasmic mtDNA in flight muscles, and show that stimulation of autophagy, or a decrease in mitofusin, promotes clearance of mutant mtDNA.

Mitochondria are membrane-bound organelles present in many copies in most eukaryotic cells. The circular mitochondrial genome (mtDNA) encodes multiple tRNAs, rRNAs and polypeptides necessary for oxidative phosphorylation, which generates the bulk of ATP in most cells. Individual mitochondria contain multiple copies of mtDNA, each of which is packaged into a structure known as a nucleoid, with primarily a single mtDNA per nucleoid^1^. This multiplicity of genomes per cell, in conjunction with mtDNA's high mutation rate and limited repair capacity, often results in cells carrying mtDNA of different genotypes, a condition known as heteroplasmy. Recent studies suggest that 90% of individuals have some level of heteroplasmy, with 20% harbouring heteroplasmies that are implicated in disease^2^,^3^. If the frequency of such a mutation reaches a threshold, pathology results^4^,^5^. High frequencies of deleterious mutant mtDNA result in severe maternally inherited syndromes^4^,^5^,^6^,^7^. Heteroplasmy for deleterious mtDNA can also arise in somatic tissues during development and in adulthood. It accumulates throughout life, and is thought to contribute to diseases of aging that include neurodegeneration, metabolic disorders, cancer, heart disease and sarcopenia^8^,^9^. These observations emphasize the importance of devising ways to reduce heteroplasmy *in vivo*.

Mitochondria-targeted site-specific nucleases, such as restriction enzymes^10^,^11^, engineered zinc-finger nucleases^12^,^13^ and transcription activator-like effector nucleases^14^,^15^, provide one way to decrease the levels of heteroplasmy. In this approach, a site-specific nuclease is engineered so as to bind and cleave a specific mutant version of the mtDNA genome, promoting its selective degradation. This approach has recently been used to decrease the levels of heteroplasmy in patient-derived cell lines^14^, in oocytes and in single cell embryos^15^. However, these methods are likely to be challenging to implement in the adult^11^, as the nuclease being expressed is a non-self protein; many cells must be targeted without off target cleavage effects; and individuals may be heteroplasmic for multiple deleterious mutations. Here we seek to promote cell biological processes that normally regulate mtDNA quality as an alternative approach to decreasing heteroplasmy in adults.

Mitophagy serves as a form of quality control that promotes the selective removal of damaged mitochondria. In one important pathway, dysfunctional mitochondria are eliminated through a process dependent on PTEN-induced putative kinase 1 (PINK1) and Parkin, loss of which lead to familial forms of Parkinson's disease. In this pathway, mitochondrial membrane depolarization, which occurs in response to mitochondrial dysfunction of various sorts, results in stabilization of the kinase PINK1 on the outer mitochondrial membrane^16^. PINK1 recruits multiple autophagy adaptors^17^ and the cytoplasmic E3 ubiquitin ligase Parkin^18^,^19^, which ubiquitinates and promotes the degradation of Mitofusins^19^,^20^,^21^, mitochondrial outer membrane proteins essential for outer mitochondrial membrane fusion^22^, thereby inhibiting re-fusion of dysfunctional mitochondria with the network. Parkin also ubiquitinates a number of other proteins^23^,^24^. These events lead to the recruitment of autophagosomal membranes, which sequester the defective mitochondria and deliver it to the lysosome for degradation.

An important question is whether mitophagy, which acts at the level of individual mitochondria, contributes to quality control at the level of mtDNA—the selective removal of mutant mtDNA within the cell as a whole. Several observations suggest that *PINK1/parkin*-dependent mitophagy may participate in such a process. First, the frequency of a deleterious allele in a heteroplasmic mammalian cell line can be reduced if cells are cultured for long periods of time in the presence of Parkin^25^, decreased membrane potential^25^,^26^ and/or stimulation of autophagy^26^,^27^. These results are intriguing, but because the experiments occur over many cell cycles, stochastic segregation of mitochondrial genomes during division, coupled with increased survival and/or proliferation of cells with an increased fraction of wild-type mtDNA, may contribute to the decreased mutant mtDNA load. Second, in lines of *Caenorhabditis elegans* heteroplasmic for an mtDNA deletion mutant, loss of Parkin (but not PINK1) results in increased levels of heteroplasmy^28^. These results also support a role for Parkin in mtDNA quality control, but they leave the nature of the selection event unclear, and whether it occurs during germline transmission and/or in somatic cells, during development and/or in the adulthood. Finally, dopaminergic neurons from mice expressing a proofreading-deficient mtDNA polymerase and wild type for *parkin*, have a spectrum of mtDNA mutations that includes fewer predicted pathogenic variants than are observed in neurons from proofreading-defective mice that lack Parkin^29^. This also is consistent with models in which Parkin promotes the elimination of some mtDNA genotypes. However, whether these events occur during development or during adulthood, and the nature of the selection event is unknown and difficult to study in a system in which mtDNA mutations are induced through random DNA polymerase errors throughout life.

Regardless, the fact that mutant mtDNA accumulates in individuals wild type for *PINK1* and *parkin* during aging, and in affected tissues in inherited maternal mitochondrial disease, indicates that if PINK1- and Parkin-dependent mitophagy and/or other pathways promote mtDNA quality control, they are often not active or effective. To identify ways of reducing the mutant mtDNA load in somatic tissues, systems are needed in which a specific deleterious heteroplasmy can be induced *in vivo* and followed over time, ideally in post-mitotic cells so as to eliminate potential confounding effects associated with stochastic segregation during cell division, and differential cell proliferation and/or cell death. Current *in vivo* models are cumbersome and limited, as they require the use of *Caenorhabditis elegans* heteroplasmic for a deletion^28^,^30^, or introduction of the cytoplasm from animals homoplasmic^31^,^32^,^33^,^34^, or cells heteroplasmic^35^ for pre-existing mutant mtDNA genotypes, into early embryos of a different genotype. Here we describe the generation and use of a transgene-based system of heteroplasmy in post-mitotic muscle to identify conditions that result in the selective removal of mutant mtDNA.

## Results

### Creation of heteroplasmy for a lethal mtDNA deletion

**Figure 1:**
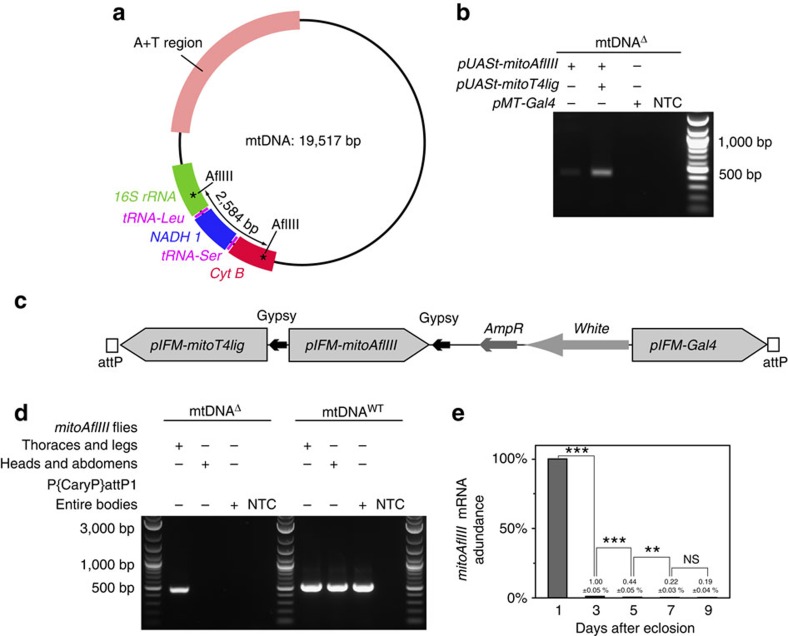
Engineered heteroplasmy for an mtDNA deletion (mtDNA^Δ^) in Drosophila. (**a**) Map of *Drosophila melanogaster* mtDNA showing the genes affected following cleavage and re-ligation at two AflIII restriction enzyme cleavage sites. (**b**) Co-expression of *mitoAflIII* and *mitoT4lig* in Drosophila S2 cells cause an increase in mtDNA^Δ^ levels. (**c**) Transgenesis construct for co-expression of *mitoAflIII*, *mitoT4lig* and *Gal4* under the control of the *Flightin* indirect flight muscle (IFM)-specific promoter in Drosophila. (**d**) Left panel: mtDNA^Δ^ is detected by PCR in the thorax, but not the head and abdomen of P{*pIFM-mitoAflIII*, *pIFM-mitoT4lig*, *pIFM-Gal4*}attP1 (*mitoAflIII*) flies. mtDNA^Δ^ is undetectable in P{CaryP}attP1 flies, which were used for a site-specific transgenesis. Right panel: mtDNA^WT^ (the presence of which results in a similarly size band in PCR) is present in all samples other than the no-template control (NTC). (**e**) The abundance of *mitoAflIII* mRNA falls precipitously after day one post eclosion. Bars depict mean±1 s.d. Statistical significance was calculated using Student's *t-*test with unequal variance. ^NS^*P*>0.05, **P<0.01 and ***P<0.001. NS, not significant.

To create a transgene-based model of a heteroplasmy that removes essential genes (lethal when homoplasmic), we first generated a mitochondria-targeted version of the restriction enzyme AflIII (mitoAflIII), which is predicted to cut Drosophila mtDNA twice, creating a 2,584 bp deletion (mtDNA^Δ^) that removes or disrupts multiple genes (Fig. 1a). S2 cells transiently transfected with mitoAflIII are heteroplasmic for mtDNA^Δ^ (Fig. 1b). The frequency of heteroplasmy was further increased following co-expression with mitochondria-targeted T4 DNA ligase (mitoT4lig) (Fig. 1b), presumably because T4 DNA ligase expression limits the degradation of AflIII cut linear products by promoting their re-ligation and re-cutting until base insertions or deletions result in products that lack AflIII sites (Supplementary Table 1).

To explore the fate of mtDNA^Δ^ *in vivo*, we sought to generate transgenic flies that specifically create mtDNA deletions in a post-mitotic tissue, so as to be able to isolate effects on mtDNA quality control from those due to differential segregation during proliferation. The Drosophila indirect flight muscle (IFM) is a non-essential, adult-specific, post-mitotic and energy-intense tissue containing a high density of mitochondria. It also shows strong and consistent phenotypes in response to the loss of genes involved in mitochondrial quality control, such as *PINK1* and *parkin* mutants^36^,^37^,^38^,^39^,^40^. We expressed mitoAflIII and mitoT4 DNA ligase under the control of an adult IFM-specific promoter (pIFM)^41^ derived from the *flightin* gene, and using the UAS–GAL4 system. While the expression of mitoAflIII and T4 DNA ligase under the control of ubiquitous or neuron-specific GAL4 drivers resulted in 100% lethality, indicating the highly deleterious nature of the deletion, as would be expected for organism-wide defects in mitochondrial function (Supplementary Table 2), expression of mitoAflIII and mitoT4lig under pIFM control resulted in viable flies with high levels of tissue-specific heteroplasmy (see below). Flies that carry these transgenes and pIFM-Gal4 (P{*pIFM-mitoAflIII, pIFM-mitoT4lig, pIFM-Gal4*}attP1) were used in subsequent experiments, and are hereafter referred to as *mitoAflIII* flies (Fig. 1c–e). pIFM-driven *mitoAflIII* messenger RNA (mRNA) expression is high at day 1 post adult eclosion, and then decreases markedly (>100 ×) by day 3, and onwards (Fig. 1e). MitoAflIII protein levels presumably follow a related time course, though a lack of antibodies to this protein prevented us from testing this directly. Thus, pIFM-driven expression provides a pulse of *mitoAflIII* expression during early adulthood, the consequences of which can then be followed over time.

### Quantification of mtDNA species

**Figure 2:**
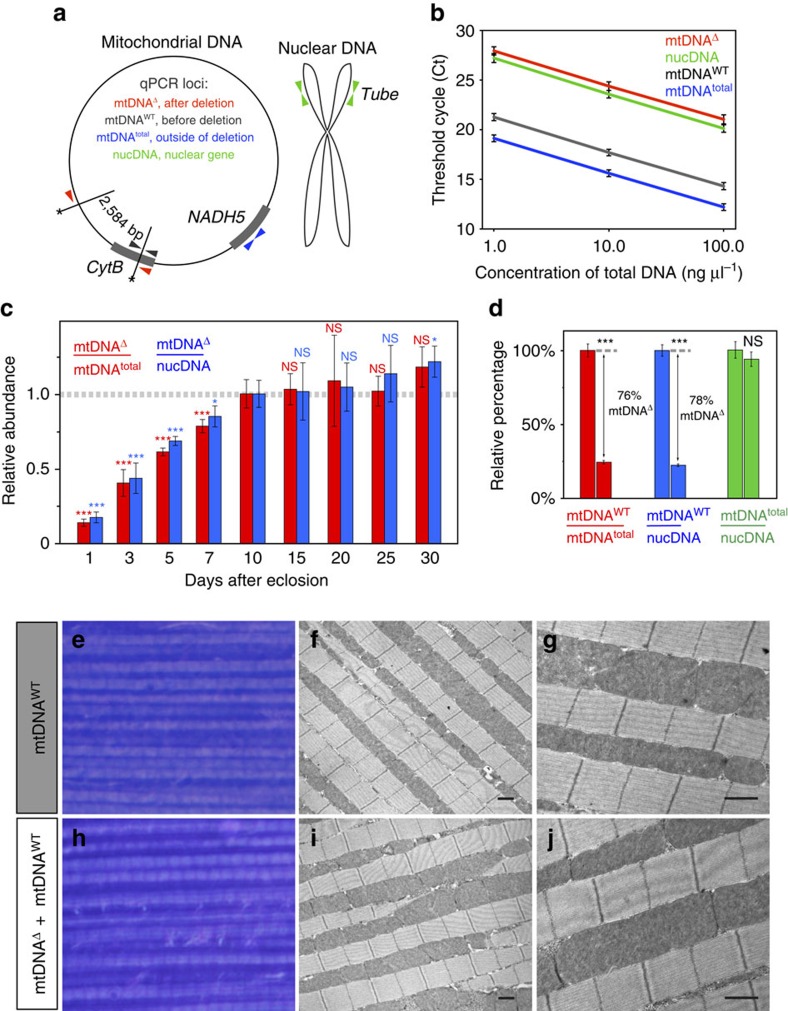
qPCR quantification of mtDNA species and effects of heteroplasmy on muscle structure. (**a**) Positions and orientations of qPCR primers are indicated by arrows. (**b**) Four standard curves connecting three average Ct values (±1 s.d.) for each primer set. (**c**) The amount of mtDNA^Δ^ in the IFMs of *mitoAflIII* flies increases through the first 10 days after eclosion and stabilizes afterwards. Relative amounts of mtDNA^Δ^ were estimated using qPCR normalized to the levels observed in 10-day-old flies, which were assigned the value of 1. Minimum four biological replicates were quantified for each time point. Statistical significance is calculated relative to the values in 10-day-old flies. (**d**) Quantification of mtDNA forms in flight muscle from 10-day-old P{*pIFM-Gal4*}attP1 (wild type, left bar of each pair) and mtDNA^Δ^ -bearing P{*pIFM-mitoAflIII, pIFM-mitoT4lig, pIFM-Gal4*}attP1 (*mitoAflIII,* right bar of each pair) flies. mtDNA^WT^ depletion among all mtDNA molecules (mtDNA^total^) corresponds to emergence of mtDNA^Δ^ molecules: that is, mtDNA^total^−mtDNA^WT^=mtDNA^Δ^. Bars depict mean±1 s.d. (**e**,**h**) Toluidine blue staining, and (**f**,**g**,**i**,**j**) transmission electron micrographs of indirect flight muscles of 10-day-old flies. Wild-type (**e**–**g**) and *mitoAflIII* (**h**–**j**) flies have similar muscular ultrastructure. Scale bars, 1.0 μm. Statistical significance was calculated using Student's *t-*test with unequal variance. ^NS^*P*>0.05, *P<0.05 and ***P<0.001. NS, not significant.

We used two complementary approaches to quantify wild type and deletion-bearing mtDNA in the IFMs, quantitative PCR (qPCR) and direct visualization *in situ*, using target-primed rolling-circle amplification (tpRCA). For qPCR, four loci were amplified from IFM DNA (Fig. 2a): mtDNA common to wild type and deletion genomes, *mt:NADH5* (mtDNA^total^); mtDNA that spans one of the AflIII sites, and which is therefore specific to wild-type mtDNA (mtDNA^WT^); mtDNA that spans the deletion, and is therefore specific to the deletion (mtDNA^Δ^); and a nuclear locus, *3R:tub*. Amplification efficiencies of these primers were similar over three orders of magnitude of DNA concentration (Fig. 2b). Quantification of mtDNA^Δ^ was done relative to both total mtDNA (mtDNA^total^) and nuclear DNA (nucDNA). Both approaches gave similar results, with the fraction of mtDNA^Δ^ increasing through the first 10 days of adulthood to ∼76% of mtDNA^total^, and then remaining stable thereafter (Fig. 2c,d). The increase in fraction of mtDNA^Δ^ over time may simply reflect the kinetics of cleavage and re-ligation of the mtDNA genome. Competitive replication advantage of the smaller deletion-bearing genome may also participate, a possibility that remains to be explored.

**Figure 3:**
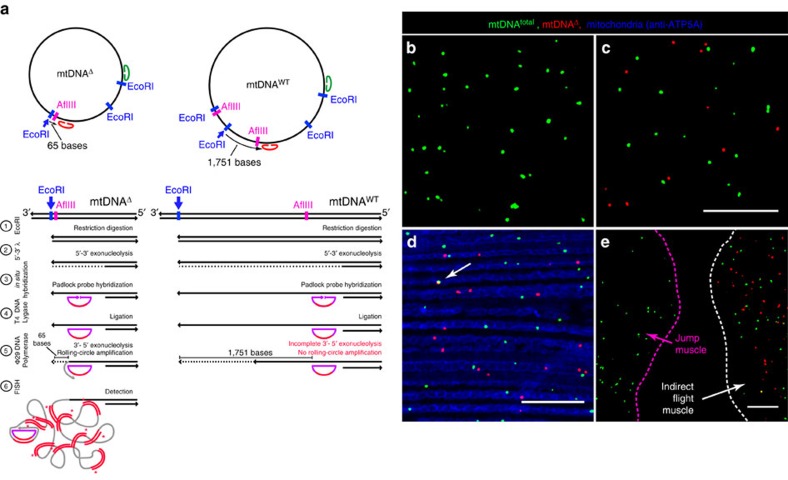
Rolling circle-based quantification of mtDNA^total^ and mtDNA^Δ^. (**a**) Strategy for visualizing mtDNA^Δ^ and total mtDNA *in situ*, using padlock probes and target-primed rolling-circle amplification (tpRCA). (**b**) P{*pIFM-Gal4*}attP1 (wild type) IFMs reacted with probes specific for mtDNA^Δ^ (red) and mtDNA^total^ (green) contain only green dots. (**c**–**e**) IFMs from P{*pIFM-mitoAflIII, pIFM-mitoT4lig, pIFM-Gal4*}attP1 (*mitoAflIII*) flies contains red and green dots. (**d**) IFMs from *mitoAflIII* flies stained with anti-ATP5A (blue) show mitochondrial localization of red and green dots. Co-localization between fluorophore-tagged mtDNA^Δ^ and mtDNA^total^ was seen only rarely (white arrow in **d**), reflecting the fact that tpRCA labels only a small fraction of nucleoids^42^. (**e**) Tissue from *mitoAflIII* flies containing IFM and the neighbouring jump muscle (Supplementary Fig. 1). Red and green dots are observed only in the IFMs, in which AflIII and T4 ligase are expressed. Scale bar, 10 μM (**c**–**e**).

As a mechanistically distinct approach to mtDNA species quantification, we imaged mtDNA^Δ^ and mtDNA^total^ *in situ*, adapting the approach of Larsson *et al.*^42^, utilizing target-primed, tpRCA. In brief, two different oligonucleotide probes, one designed to provide a signal when bound to mtDNA^Δ^ and one designed to provide a signal when bound to any mtDNA (mtDNA^total^), were circularized after hybridization to target sequences *in situ*. These circles provide templates for localized amplification mediated by Φ29 DNA polymerase, using a nearby target DNA 3′-end as a primer. The 3′-end for both probes was generated by EcoRI cleavage. mtDNA^Δ^ can be specifically identified using this approach because the ability of a 3′-end in fixed tissue to act as a primer drops markedly with distance from the padlock probe^42^. Therefore, Φ29-dependent amplification from the circularized mtDNA^Δ^ probe only occurs when one of the EcoRI sites has been brought near the post-cleavage AflIII site, located adjacent to the mtDNA^Δ^ probe hybridization site. Conversely, amplification from the circularized mtDNA^total^ probe occurs when a different 3′-end is utilized, generated by EcoRI cleavage at a distinct site (Fig. 3a; Methods).

As expected, flight muscle from wild-type flies labels only with the mtDNA^total^ probe (Fig. 3b), while flight muscle from 10-day-old *mitoAflIII* flies labels with both mtDNA^total^ and mtDNA^Δ^ probes (Fig. 3c). Both signals co-localize with mitochondria, as visualized with an antibody that stains the mitochondrial matrix (Fig. 3d). Fluorescent signals rarely co-localize (Fig. 3d) because tpRCA labels only a small fraction of mtDNA nucleoids^42^. mtDNA^Δ^ labelling was only detected in IFMs of *mitoAflIII* flies, but not in the neighbouring jump muscle, in which *mitoAflIII* is not expressed (Fig. 3e; Supplementary Fig. 1). Importantly, the fractions of mtDNA^total^ consisting of mtDNA^Δ^ determined using qPCR and tpRCA were comparable once a correction factor was applied to the tpRCA data so as to take account of the fact that the probability of RCA is dependent on the distance of the padlock probe to the 3′-end used as primer^42^ (Methods; Supplementary Table 3). Finally, we note that tpRCA is not used to estimate the total tissue levels of mtDNA, since only a fraction of nucleoids are labelled. Estimates of total muscle mtDNA (using nucDNA as a point of comparison, Supplementary Table 3) are derived from qPCR analysis.

### Effects of mtDNA^Δ^ heteroplasmy on IFM function

To explore the effects of high levels of heteroplasmy in more detail, we examined flight muscle from 10-day-old wild-type and *mitoAflIII* flies using light microscopy and transmission electron microscopy. Muscle morphology was similar for both genotypes (Fig. 2e–j). In particular, at the transmission electron microscopy level mitochondria were of similar size and abundance, with intact cristae. We also tested flight muscle function using an assay commonly used to quantify flight performance in Drosophila^43^. Wild-type and *mitoAflIII* flies showed similar abilities in this assay (Supplementary Fig. 2). These results suggest that high levels of heteroplasmy do not result in dramatic defects in function, and are consistent with other observations showing high levels of heteroplasmy for a deleterious mutation in phenotypically normal animals, including Drosophila^34^ and humans^2^,^44^,^45^.

### Autophagy promotes selective removal of mtDNA^Δ^

**Figure 4:**
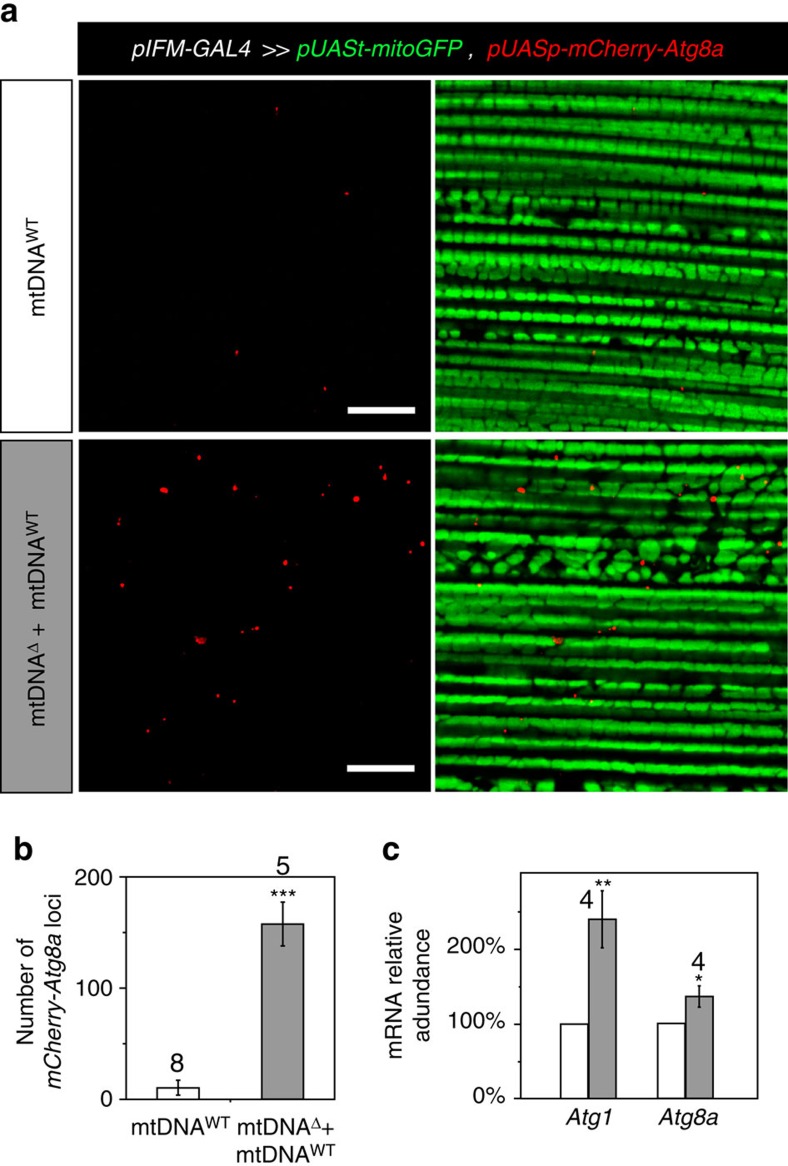
Evidence for induction of autophagy in response to mtDNA^Δ^. (**a**) IFM from a 3-day-old wild-type animal expressing mitochondria-localized *GFP* (*mitoGFP*; green) and *mCherry-Atg8a* (red) under the control of *pIFM-GAL4* (top panels), and a *mitoAflIII*-expressing, mtDNA^Δ^-bearing animal (lower panels). Scale bar, 10 μM. (**b**) Histogram quantifying abundance of mCherry-Atg8A associated structures in wild-type and mtDNA^Δ^-bearing IFMs. (**c**) Relative mRNA abundance of *Atg1* and *Atg8a* in wild-type IFMs (mtDNA^WT^), and IFMs from *mitoAflIII* animals containing mtDNA^Δ^ (mtDNA^Δ^+mtDNA^WT^), at 3 days post eclosion. Bars depict mean±1 s.d. The number of biological replicate is indicated above the corresponding bars. Statistical significance was calculated using Student's *t-*test with unequal variance. *P<0.05, **P<0.01 and ***P<0.001.

To explore roles for autophagy in mtDNA quality control, we expressed *mCherry-Atg8a*^46^ in the IFMs of otherwise wild-type animals, with or without *mitoAflIII*. Atg8 is a cytosolic ubiquitin-like protein that is required for the formation of autophagosomal membranes, becoming covalently linked with them during autophagosome formation. Autophagosome formation can therefore be visualized as the formation of discrete mCherry-Atg8a puncta^46^,^47^. Few Atg8a-positive structures were observed in IFMs from 3-day-old widl-type flies (Fig. 4a). In contrast, IFMs from similarly aged *mitoAflIII* flies showed a large number of positive structures, an increase of roughly 50 × (Fig. 4a,b). Expression of endogenous *Atg8a* was also upregulated in *mitoAflIII* flies, as was that of *Atg1* (Fig. 4c), a master upstream activator of autophagy.

**Figure 5:**
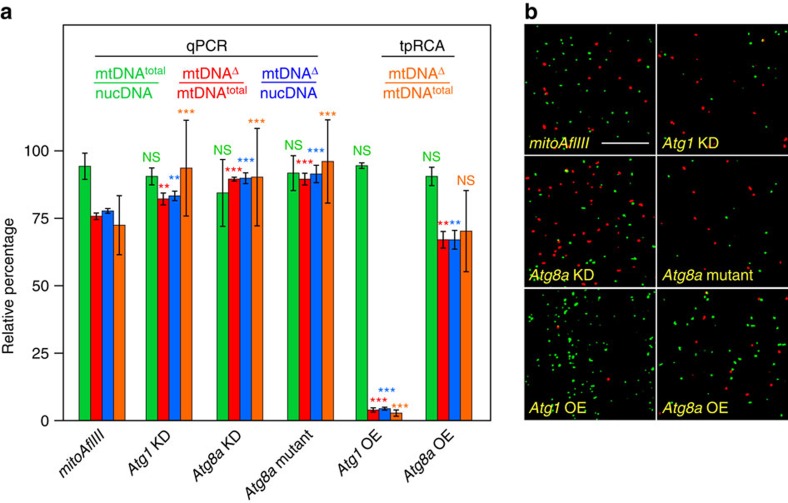
*Atg1* and *Atg8a* promote selective removal of mtDNA^Δ^. All data presented are derived from Supplementary Table 3. (**a**) Relative abundance of different mtDNA species determined using qPCR and tpRCA. Bars depict mean±1 s.d. Supplementary Table 3 shows numbers of biological replicates. (**b**) Images from flight muscle of the indicated genotypes, hybridized for tpRCA as in Fig. 3. Scale bar, 10 μM. Statistical significance is estimated as compared with levels of the corresponding species in *mitoAflIII* flies using Student's *t-*test with unequal variance. ^NS^*P*>0.05, **P<0.01 and ***P<0.001. Note that number of nucleoids labelled in each sample in **b** is not reflective of total muscle mtDNA levels, which are estimated in **a** using qPCR and comparisons with nuclear DNA (mtDNA^total^/nucDNA). NS, not significant.

**Figure 6:**
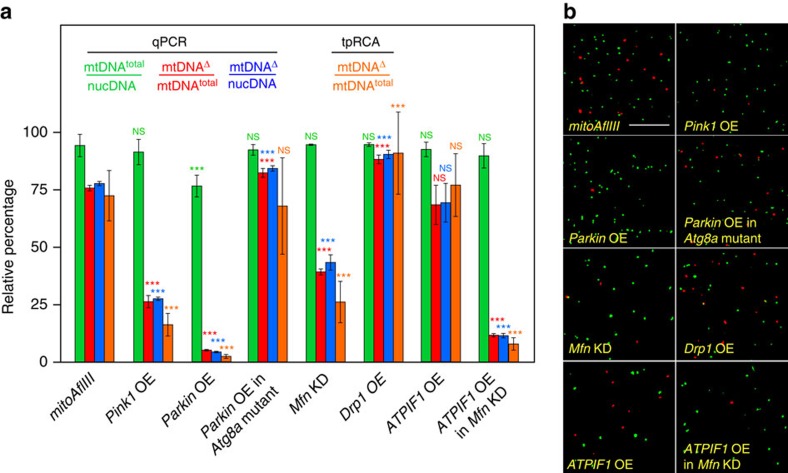
Effects of manipulating PINK1/Parkin and Mfn and ATPIF1 on mtDNA^Δ^ levels. (**a**) Relative abundance of different mtDNA species determined by qPCR and tpRCA. Bars depict mean±1 s.d. (**b**) Images from flight muscle of the indicated genotypes, hybridized for tpRCA as in Fig. 3. Scale bar, 10 μM. Statistical significance is estimated as compared with levels of the corresponding species in *mitoAflIII* flies using Student's *t-*test with unequal variance. ^NS^*P*>0.05 and ***P<0.001. (**a**,**b**) Note that all data derives from Supplementary Table 3. Part of this, the *mitoAflIII* qPCR data, and the *mitoAflIII* tpRCA panel, are presented here and in Fig. 5 to facilitate comparison across genotypes, in particular with P{*pIFM-mitoAflIII, pIFM-mitoT4lig, pIFM-Gal4*}attP1 (*mitoAflIII*) flies in an otherwise wild-type background. Note that as in Fig. 5, total number of nucleoids labelled in each sample in **b** is not reflective of total muscle mtDNA levels, which are estimated using qPCR. NS, not significant.

If autophagy represents an attempt to remove dysfunctional mitochondria that carry mutant mtDNA, decreasing the levels of Atg1 and Atg8a should lead to an increase in mtDNA^Δ^. In considering this and other questions (Figs 5 and 6), we characterized mtDNA species at day 10 so as to provide ample time for mtDNA quality control following the pulse of pIFM-driven AflIII and T4 DNA ligase expression (Fig. 1e). For simplicity, in the text below, we refer only to measurements of mtDNA calculated from mtDNA^Δ^/mtDNA^total^ using qPCR, though other methods gave similar results (Figs 5 and 6; Supplementary Table 3). Comparisons of heteroplasmy levels across all genotypes are made with respect to those of mitoAflIII flies (Supplementary Table 3). Consistent with the above hypothesis, RNAi of *Atg1* resulted in an increase in the frequency of mtDNA^Δ^ (from 76±1 to 82±2%), as did knockdown or mutation of *Atg8a* (from 76±1% to 89±1% or 89±2%, respectively; Fig. 5; Supplementary Figs 3 and 4). Conversely, overexpression of *UAS-Atg1* or *UAS-Atg8a* under pIFM-Gal4 control resulted in the specific loss of mtDNA^Δ^ (from 76±1 to 4±1% or 67±3%, respectively; Fig. 5). This effect was particularly marked in the case of *Atg1* expression, which resulted in the removal of 95% of the mutant genomes. The stronger effects of Atg1 overexpression may reflect the fact that Atg1 is an upstream activator of autophagy^48^, expression of which is sufficient to drive autophagy^49^. In contrast, Atg8 functions downstream of autophagy activation; it serves as a protein–protein interaction platform and structural component of the autophagosome, and its expression levels regulate autophagosome size^48^,^50^. *Atg1* is regulated in a number of ways *in vivo*. In one important pathway, the *mTOR* complex negatively regulates *Atg1* in nutrient-rich conditions^51^. Interestingly, exposure of flies raised on normal (nutrient-rich) media to the *mTOR* inhibitor rapamycin also resulted in a modest decrease in mtDNA^Δ^ frequency (Supplementary Fig. 5). Together, these results suggest that autophagy participates in the removal of mtDNA^Δ^ in Drosophila IFMs. This process is inefficient in wild-type animals, but can be stimulated through further activation of autophagy.

### PINK1 and Parkin promote autophagic removal of mtDNA^Δ^

PINK1 and Parkin were first shown to function in a common pathway in Drosophila^38^,^39^,^40^,^52^, with the loss of either protein resulting in marked phenotypes in multiple tissues. IFM phenotypes include extensive cell death and severe defects in mitochondrial integrity and function^36^,^37^,^38^,^39^,^40^. Results of mass spectrometry show that the normal turnover of a number of mitochondrial proteins in adult Drosophila is both Parkin and autophagy dependent, suggesting that PINK1 and Parkin promote mitophagy on an ongoing basis in wild-type animals^53^. However, whether they promote mtDNA quality control has not been examined. We characterized the consequences of PINK1 and Parkin overexpression, as extensive cell death precludes study of loss-of-function mutants^36^,^37^,^38^,^39^,^40^. Overexpression of PINK1 resulted in a large decrease in mtDNA^Δ^ frequency, from 76±1 to 26±3%. Overexpression of Parkin resulted in an even larger decrease in mtDNA^Δ^ frequency, from 76±1 to 5±0.3%, which was associated with a modest decrease in mtDNA^total^ (Fig. 6). Importantly, IFMs from *mitoAflIII* double mutants in which Parkin was overexpressed in an *Atg8a* mutant background had an increased frequency of mtDNA^Δ^ as compared with *mitoAflIII* flies (82±2% versus 76±1%; Fig. 6). These levels are similar to those observed when *mitoAflIII* is expressed in an *Atg8a* mutant background (Fig. 5). These results suggest that PINK1 and Parkin act through autophagy to promote the removal of mtDNA^Δ^, and that expression level of these proteins is rate limiting for mtDNA quality control in otherwise wild-type animals.

### Decreased Mfn promotes selective removal of mtDNA^Δ^

Because mitochondria are only tested for quality when isolated from the network^54^, the rate of re-fusion, which determines the half-life of isolated fragments, is expected to play an important role in quality control. This is because mutant-bearing fragments can only be detected as such if their half-life is long as compared with the time needed for depolarization and tagging as defective, which will itself depend on the nature of the underlying genetic defect and half-life of wild-type versions of the encoded products provided through complementation^55^,^56^,^57^. A prediction of this model is that decreasing the levels of *mfn* should promote selective mutant mtDNA removal by increasing the half-life of isolated mitochondrial fragments, and/or by lowering the threshold amount of Mfn that needs to be degraded by the limiting amounts of PINK1 and Parkin available (Fig. 6) to inhibit re-fusion. We used RNA interference to decrease Mfn in the IFMs to levels sufficient to bring about a decrease in mitochondrial size and rescue other *PINK1* and *parkin* loss-of-function phenotypes^58^,^59^. Consistent with the above hypothesis, this resulted in a large decrease in deletion levels (from 76±1 to 39±1%; Fig. 6). The converse experiment, increasing levels of *mfn*, could not be carried out as this results in extensive cell death^41^. Interestingly, overexpression of the pro-fission protein Drp1 at levels which also rescue *PINK1 and parkin* loss-of-function phenotypes and bring about a similar decrease in mitochondrial size^58^ did not result in a decrease in deletion levels, with levels being instead somewhat increased (from 76±1 to 88±2%; Fig. 6). Why increased Drp1 results in an increased frequency of deletions requires further exploration. One possible mechanism suggested by modelling studies is that increased levels of Drp1, in conjunction with wild-type levels of Mfn, which our results suggest (Fig. 6) are higher than optimal for efficient mtDNA^Δ^ removal, leads to more rapid cycles of fusion and fission, promoting content mixing and complementation, thereby defeating quality control^55^,^56^,^57^.

### Synergy between ATPIF1 and Mfn in reducing mtDNA^Δ^

Finally, in order for mitochondria isolated from the network to be tested for mtDNA quality, it is not enough that they remain isolated from the network. They must also present an ‘honest signal' as to genome dysfunction, typically in the form of depolarization of the mitochondria in which these genomes reside. Presentation of such a signal can be inhibited through the action of other regulators of mitochondrial physiology. For example, mitochondria depolarization can lead to reversal of the F^0^F^1^ ATP synthase, which then pumps protons into the matrix, restoring the mitochondrial inner membrane potential at the expense of cytosolic ATP. This process is regulated by the ATPIF1 protein, which inhibits ATPase activity, but not synthase activity. The importance of ATP synthase reversal in inhibiting mitophagy is suggested by observations of mammalian cell cultures treated with a mitochondrial uncoupler^60^. In the presence of wild-type levels of ATPIF1 mitochondria undergo depolarization and recruitment of Parkin. However, if they are depleted of ATPIF1, the mitochondrial membrane fails to depolarize or recruit Parkin^60^. To explore roles that ATPIF1 plays in mtDNA quality control, we expressed Drosophila ATPIF1 in *mitoAflIII* flies. This alone had no significant effect on mtDNA^Δ^ levels. Interestingly, however, expression of ATPIF1 in the presence of RNAi of *mfn* resulted in a synergistic decrease in mtDNA^Δ^ levels, from (from 76±1 to 12±1%), as compared with RNAi of *mfn* alone (from 76±1 to 39±1%; Fig. 6). These observations suggest that ATP synthase reversal can antagonize mtDNA quality control, and that drugs that mimic ATPIF1 activity by inhibiting ATPase activity but not ATP synthase activity^61^,^62^ may be useful in promoting quality control.

## Discussion

We have generated a transgene-based model of mtDNA heteroplasmy in an adult post-mitotic tissue, the adult flight muscle. The high levels of heteroplasmy (∼76%) did not interfere with flight muscle function, making this a useful system for exploring mtDNA quality control in a quantitative manner. It may also prove useful as a system in which to identify compensatory mechanisms that buffer muscle function from effects caused by high levels of heteroplasmy. Finally, we demonstrate that the load of deleterious mtDNA can be decreased through several different interventions. Genetic and chemical screens using such a model should prove useful in identifying molecules that can cleanse tissues of a deleterious genome, via known and unknown mitochondrial quality control pathways. The many tools for regulated spatial and temporal control of gene expression in Drosophila will allow such screens to be carried out in a variety of tissues and environmental contexts, including aging^63^.

Our results show that adult muscle has a significant but limited ability to remove mutant mtDNA utilizing genes required for autophagy, and that mtDNA^Δ^ removal can be greatly stimulated in several ways: by limiting the ability of mitochondrial fragments to re-fuse with the network (decreasing Mfn levels), by limiting their ability to undergo repolarization through ATP synthase reversal (ATPIF1 expression), by increasing the tagging of mtDNA^Δ^-bearing fragments (increasing PINK1 or Parkin levels), and by increasing the frequency with which these tagged fragments are degraded (activation of autophagy). These observations have important implications for new therapies for mitochondrial disease and diseases of aging.

Our observation that wild-type levels of key proteins are not set to maximize mtDNA quality control provides a possible explanation for why mutant mtDNA accumulates in multiple tissues during normal aging: the components are required at different levels in other more immediate contexts. Thus, mitochondrial dynamics are often driven to promote rapid cycles of fusion and fission so as to increase network connectivity, ATP production and/or calcium handling^22^,^64^,^65^. These conditions are predicted to facilitate mutant mtDNA maintenance or accumulation^55^,^56^,^57^. Similarly, levels of ATPIF1 may primarily reflect the need to buffer mitochondrial membrane potential from transient changes associated with muscle contraction and/or mediate metabolic reprogramming and responses to stress^66^. PINK1 and Parkin also play a number of non-mitophagic roles in Drosophila and mammalian systems, which may be costly if activated continuously^67^,^68^,^69^. Continuous activation of autophagy leads to cell death, tissue catabolism and changes in metabolism, and is normally tightly regulated at multiple levels^50^. In the face of these competing cellular demands, expression tuned to maximize the removal of mutant mtDNA throughout life probably would result in significant life-history costs, particularly since defects in mtDNA quality control in somatic tissues manifest themselves primarily in later life^8^,^9^. A key question is whether occasional manipulations of cell physiology that promote mtDNA quality control, in otherwise healthy individuals, can bring about a more general ‘housecleaning' that keeps the frequency of mutant DNA below the threshold for causing cellular dysfunction in diverse tissues without incurring other organismal costs.

## Methods

### Generation of constructs

The protein sequence of AflIII restriction enzyme (protein ID: ADO24177.1) without the N-terminal *Methionine* was back translated and codon optimized for translation in Drosophila in Gene Designer 2.0 (https://www.dna20.com/resources/genedesigner). The N-terminal tag directing protein import into the mitochondrial matrix (60 amino acids from the N terminus of Drosophila *Aconitase* (CG9244), plus a C-terminal *Alanine*) was identified with the Target P 1.1 program (http://www.cbs.dtu.dk/services/TargetP/). It was then appended to the N terminus of *AflIII*, generating *mitoAflIII.* To build the *pUASt-mitoAflIII*, a *mitoAflIII* fragment was gene synthesized by DNA2.0 and cloned into pWALIUM 20 (https://plasmid.med.harvard.edu/PLASMID/GetVectorDetail.do?vectorid=500) between XbaI and NdeI sites.

A mitochondria-targeted version of T4 DNA ligase was built as follows. The coding sequence (CDS) of T4 DNA ligase was PCR amplified from *Escherichia coli* carrying T4 bacteriophage. An out of frame synthetic intron was inserted into the CDS of *T4 ligase* to facilitate bacterial cloning and manipulation. To generate *pUASt-mitoT4ligase*, the N-terminal mitochondrial targeting sequence utilized with AflIII, *T4 ligase* and two translations enhancers IVS (a small 5′-untranslated region (UTR) intron from the Drosophila *Mhc* gene, CG17927) and *p10* 3′-UTR (the terminator sequence from the *AcNPV p10* gene) were cloned into pWALIUM 20 cut at BglII and HpaI sites). To build constructs for overexpression of *parkin*, *drp1* and *Atg8a*, a *mitoT4ligase* fragment in *pUASt-mitoT4ligase* plasmid was replaced with corresponding CDSs. CDSs of *parkin*, *drp1*, *Atg8a* and *ATPIF1* were PCR amplified from Drosophila cDNA, reverse-transcribed with an oligo-dT primer from total RNA.

A three-transgene construct (*pIFM-mitoAflIII*, *pIFM-mitoT4lig* and *pIFM-Gal4*) that directs IFM-specific expression of mitochondrially targeted *AflIII* and *T4 ligase*, and the yeast transcriptional activator *Gal4*, was built in pWALIUM 20 (Fig. 1c; GenBank, KX696451). The *Flightin* promoter (*pIFM*) was PCR amplified from *Drosophila melanogaster* genomic DNA, using primers 5′-CGTTCCCGTGATAGAGTAACGGTTCCT-3′ and 5′-CAGCTAAAACTAGGACATTGGGTCCACTG-3′, 543 bases 5′ to the start site of transcript variant B of the *Flightin* gene (CG7445) and 23 bases 5′ of its 5′-UTR, respectively. The fragment between the two gypsy insulators in pWALIUM 20 was removed with PstI and HpaI. *pIFM*, *IVS*, *mitoAflIII* and *p10* 3′-UTR fragments were then ligated together. The resulting plasmid was then cut with StuI and *pIFM-mitoT4lig* was cloned, 5′–3′, from the gypsy insulator towards the attP site. Finally, *pIFM-Gal4* was cloned, 5′–3′, into the XhoI site, from the *White* gene towards the attP site (Fig. 1c).

### Transfection of Drosophila S2 cells

Drosophila S2 cells were maintained using standard protocols (Invitrogen, #R690-07). Transient transfections were performed using FuGene HF (Roche) with the following plasmids: *pUASt-mitoAflIII*, *pUASt-mitoT4lig*, *pMT-Gal4* and *pJFRC81* and *pCaSpeR-hs*. *pJFRC81* (*pUASt-eGFP-p10*) was added to visualize the efficiency of transfection and activation; and *pCaSpeR-hs* (empty *pHsp70* vector) to control for the amount of transfected DNA. *pMT* was induced with 400 μM of CuSO_4_ 7  h after transfection. Thirty hours post transfection, DNA was extracted from S2 cells and analysed by PCR.

### PCR detection of the engineered mtDNA deletion

DNA from S2 cells and Drosophila tissues was extracted with the DNeasy Blood and Tissue kit (Qiagen). The following two primers flanking the two AflIII sites in Drosophila mtDNA were used to PCR mtDNA^Δ^: 5′-ATCATATTTGTCGAGACGTTAATTATGGTTG-3′ and 5′-GAAATGAAATGTTATTCGTTTTTAAAGGTATCTAG-3′. These primers amplify either a 481 bp product from mtDNA^Δ^ molecules or a 3,065 bp product from mtDNA^WT^ molecules. The following primers complementary to the *mt:CytB* locus, located inside the deleted fragment, were used to amplify a 498 bp product present in mtDNA^WT^: 5′-GGACGAGGAATTTATTACGGTTCATA-3′ and 5′-GTGTTACTAAAGGATTTGCTGGAAT-3′. LongAmp^TM^ *Taq* DNA polymerase (New England BioLabs, NEB), with a 65 °C elongation temperature, was used for PCR with the following conditions: (94 °C for 20 s, 51.5 °C for 13 s, and 65 °C for 25 s) × 32 cycles. PCR amplicons were sequenced directly. If sequencing showed the presence of different templates (as determined by ambiguity at or near the post-cleavage AflIII site), they were first cloned into TOPO TA vector (Invitrogen) and then multiple individual clones were sequenced.

### Drosophila strains and genetics

Flies were maintained on the standard cornmeal/soy flour/yeast fly food at 25 °C with a 12H/12H light and dark cycle. The following fly stocks were obtained for this study: *da-Gal4* (Bloomington #8641); *elav-Gal4* (Bloomington #8765); *Mef2-Gal4* (Bloomington #27390); *ey-Gal4* (Bloomington #5534 & #5535); *r4-Gal4* (Bloomington #33832); *pUASt-mitoGFP*^58^; *pUASp-mCherry-Atg8a* (Bloomington # 37750, from which Dr^1^/TM3, Ser^1^ was removed); *pUASt-Atg1*(6B) III (gift from Thomas Neufeld, University of Minnesota); *Atg1* RNAi line (Bloomington #26731); *Atg8a* RNAi line (Vienna Drosophila RNAi Center #109654); *Atg8a*^*KG07569*^/FM7c (Bloomington #14639); *pUASt-Pink1* (ref. 38); *pUASt-RNAi-Marf*^58^.

Constructs were injected at Rainbow Transgenic Flies, Inc. (http://www.rainbowgene.com). The constructs containing *pIFM-Gal4* were inserted at the P{CaryP}attP1 site on the second chromosome (Bloomington #8621), while constructs with trans-activated genes were inserted at the P{CaryP}attP2 on the third chromosome (Bloomington #8622). Transgenic flies were balanced against the same background: w^1118^; CyO/sna^Sco^ and w^1118^; TM3, Sb^1^/TM6B, Tb^1^, respectively.

### *MitoGFP* and *pUASp-mCherry-Atg8a* expression and visualization

To visualize mitochondria in the Drosophila IFMs *pUASt-mitoGFP*^58^ was recombined into two genetic backgrounds: P{*pIFM-mitoAflIII*, *pIFM-mitoT4lig, pIFM-Gal4*}attP1 and P{*pIFM-Gal4*}attP1. The two generated lines were crossed to flies carrying the marker of autophagy, *pUASp-mCherry-Atg8a*^46^. The IFMs were dissected from 3-day-old male flies and fixed for 35 min in PBS with 4% paraformaldehyde and 0.2% Triton X-100. After washing three times in PBS and 0.2% Triton X-100, samples were mounted into Vectashield mounting medium and imaged on a confocal scope (Olympus FluoView-1000). Images were acquired with a × 60 (numerical aperture=1.30) objective and 1,024 × 1,024 resolution at 12 bits per pixel. Each channel (*GFP* and *mCherry*) was scanned consecutively at 12.5 μs per pixel. Three slides were prepared and examined for each genetic group. Six images applying a twofold zoom were acquired for quantification (Fig. 4b). A higher magnification (a fourfold zoom) was used to acquire images for presentation (Fig. 4a).

### Quantification of mtDNA^Δ^ with qPCR

Aged male flies were fixed in 100% ethanol and stored at 4 °C. After fixing flies for at least 14 h, IFMs were dissected in 100% ethanol. Twenty male flies were used per replicate sample. Ethanol was then removed and the sample dried on a heatblock for 15 min at 90 °C. DNA was extracted from dried IFMs with the NucleoSpin Tissue XS kit (Macherey-Nagel). DNA concentration was measured with the NanoDrop ND-1000 (Thermo Scientific) and adjusted to 10 ng μl^−1^.

The abundance of mtDNA^Δ^ was quantified relative to both mtDNA^total^ and nucDNA using real-time qPCR. The relative quantification of mtDNA deletions with qPCR has previously shown to correlate well with estimates based on Southern blotting^70^. Real-time qPCR was performed with the SYBR Green I Master kit (Roche) on the LightCycler 480 real-time PCR system (Roche). An elongation step of PCR was done at 65 °C for 25 s. Amplifications of four loci were assessed with qPCR: *mt:cytB* (specific for mtDNA^WT^), *mt:AflIII*^Δ^ (specific for mtDNA^Δ^), *mt:NADH5* (mtDNA^total^) and *nuc:Tube* (nucDNA single-copy locus; CG10520; Supplementary Table 4; Fig. 2a). The first two loci assessed the amounts of mtDNA^WT^ and mtDNA^Δ^, respectively; the second two loci provided different references for normalization of the amount of mtDNA^Δ^ (ΔCt). Melting curve analysis did not identify any primer-dimer peaks in melting profiles. To estimate the dynamic range, efficiency and sensitivity for each primer set, we constructed a standard curve covering three orders of magnitude of total DNA concentration. DNA was extracted from the dissected IFMs of w^1118^; P{*pIFM-mitoAflIII, pIFM-mitoT4lig*, *pIFM-GAL4*}attP1*/CyO* 10-day-old male flies. The four primer sets have identical efficiencies and sensitivity though the entire range (Fig. 2b). Based on this, we applied a simple ΔΔCt quantification algorithm to measure the effect of a tested gene (OE or KD) on the level of mtDNA^Δ^. The w^1118^; P{*pIFM-mitoAflIII, pIFM-mitoT4lig*, *pIFM-GAL4*}attP1*/*+ 10-day-old male flies were the reference sample for all comparisons. At least four biological replicates were analysed for each genetic background. The calculations of ΔΔCt, fold difference and normalized percentages were performed in Microsoft Excel for Mac 2011. Equations of the ΔΔCt algorithm are presented in Supplementary Methods.

### Toluidine blue staining and transmission electron microscopy

Thoraces from 10-day-old P{*pIFM-Gal4*}attP1 (wild type) and P{*pIFM-Gal4, pIFM-mitoAflIII, pIFM-mitoT4lig*}attP1 (*mitoAflIII* flies) male flies were dissected, fixed in paraformaldehyde/glutaraldehyde, post-fixed in osmium tetraoxide, dehydrated in ethanol and embedded in Epon. Blocks were cut to generate 1.5-μm-thick sections using a glass knife, or 80-nm-thick sections using a diamond knife on a microtome (Leica, Germany). Toluidine blue was used to stain 1.5-μm-thick tissue sections. Thin sections (80-nm thick) were stained with uranyl acetate and lead citrate, and examined using a JEOL 100C transmission electron microscope (UCLA Brain Research Institute Electron Microscopy Facility). At least six thoraces were examined for each genotype.

### Flight assay

Flight performances of *mitoAflIII* and wild-type 10-day-old male flies were compared using the ‘cylinder drop assay'^43^. Five groups of 30–50 flies of each type were introduced into the top of a 500-ml graduated cylinder whose internal walls were coated with paraffin oil. Wild-type flies quickly initiate horizontal flight, striking the wall close to the entry level, whereas poor fliers land at lower levels or at the bottom of the cylinder. The distribution of heights at which the flies stuck in the oil reflects their flying performance.

### Quantification of transcript abundance with RT–qPCR

The w^1118^; P{*pIFM-mitoAflIII, pIFM-mitoT4lig, pIFM-GAL4*}attP1/*CyO* (*mitoAflII*) transgenic male flies were used to quantify the transcription of *pIFM-mitoAflIII*. Five time points were used to probe the mRNA abundance of *mitoAflIII*: 1, 3, 5, 7 and 9 days post eclosion. The expression of *Atg1* and *Atg8a* gene was analysed in the same transgenic flies at day 3 post eclosion. Total RNA was extracted from the dissected thoraces and legs of flies using the mirVana miRNA isolation Kit (Ambion). To remove DNA contamination, 3 μg of total RNA was treated with the Turbo DNA-free kit (Ambion). Then, cDNA was synthesized from 500 ng of RNA with SuperScript III reverse transcriptase (Invitrogen) using an oligo-dT primer. qPCR with reverse transcription (RT–qPCR) was performed with the SYBR Green I Master kit (Roche) and the CFX96 Real-Time PCR detection system utilizing the Bio-Rad C1000.

Primers used for RT–qPCR are listed in Supplementary Table 5. We placed one primer directly on a splice junction to avoid amplification of genomic DNA. A melting curve analysis was performed to confirm the absence of any primer-dimer peak in the melting profile. Three dilution series (1/1, 1/10 and 1/100) were used to build a standard curve and estimate the dynamic range, efficiency and sensitivity for each primer set. The mRNA abundances of *mitoAflIII*, *Atg1* and *Atg8a* transcripts were normalized to *β glucuronidase* (*βGlu*) mRNA, according to the ΔCt method with an efficiency correction (Supplementary Methods). Four biological replicates were analysed per sample. mRNA abundances of *mitoAflIII* at different time points were compared with the value at 1 day post eclosion, which was given the value of 100% (Fig. 1e). Three-day-old w^1118^; P{*pIFM-GAL4*}attP1/*CyO* male flies served as a reference for estimation of changes in *Atg1* and *Atg8a* mRNA abundance in the *mitoAflII* flies.

### Target-primed tpRCA and immunochemistry

We performed target-primed RCA in whole-mount IFMs dissected from male flies using a modified version of a published method^42^. In brief, mtDNA in fixed tissue was cleaved with the restriction enzyme EcoRI and then made single stranded using λ 5′–3′ exonuclease. Two different padlock probes were then hybridized to mtDNA. One hybridization site was located near (44 bp) an EcoRI site present in widl type and mutant mtDNA (green oval in Fig. 3a). A second probe hybridizes near (66 bp) an EcoRI site that is brought close to the remaining AflIII site following mitoAflIII cleavage and re-ligation, but located 1.71 kb away from the probe binding site in widl-type mtDNA (red oval in Fig. 3a). After ligation of the padlock probes, rolling-circle amplification using Φ29 DNA polymerase was carried out, using the nearby 3′-end provided by EcoRI cleavage as a primer. This results in many rounds of amplification of the padlock probe sequence, which is detected through hybridization with fluorescently labelled probes (dpMtDNA^total^-AlexaFluor488; and dpMtDNA^Δ^-TAMRA; green and red loci, respectively; Fig. 3). mtDNA^Δ^ can be specifically identified using this approach because the ability of a 3′-end in fixed tissue to act as a primer drops markedly with distance from the padlock probe^42^, and thus only occurs when one of the EcoRI sites has been brought near the post-cleavage AflIII site (Fig. 3a, details below). We note that the efficiency of tpRCA-based labelling of nucleoids in muscle is low, as is also seen in mammalian cells^42^. Because of this, and the fact labelling efficiency can vary several fold, tpRC is not used to estimate the absolute levels of mtDNA. It can only be used to determine the fraction that is mtDNA^Δ^.

All reactions were performed inside 0.2 ml PCR tubes. At each step, we incubated samples with an enzyme or probe at 4 °C for 1 h, to allow for tissue penetration, before raising the temperature to 37 °C. The IFMs were dissected from 10-day-old flies and fixed in 4% formaldehyde in PBS with 0.3% Triton X-100 for 35 min. The fixed tissue was rinsed three times for 5 min in wash A (PBS with 0.2% Triton X-100) at room temperature (RT), heated at 75 °C for 15 min and chilled on ice for 2 min. DNA was digested using 0.5 U μl^−1^ of EcoRI HF (NEB) at 37 °C for 40 min, and rinsed three times in wash A at RT for 5 min. Hybridization target sequences for two padlock probes (Supplementary Table 6) were located either near an EcoRI site present in both mtDNA^WT^ and mtDNA^Δ^ (mtDNA^total^, green oval; Fig. 3a), or near an EcoRI site only brought near the hybridization site through creation of a deletion (mtDNA^Δ^, red oval; Fig. 3a). We applied 0.2 U μl^−1^ of the 5′–3′ λ exonuclease (NEB) at 37 °C for 15 min to make a single-stranded DNA target for complementary padlock probes.

Two 5′-end phosphorylated padlock probes served as a template for target-primed RCA: ppMtDNA^total^ and ppMtDNA^Δ^. The padlock probe hybridization site for ppMtDNA^total^ is located 44 bp upstream of one EcoRI site, while the padlock probe hybridization site for ppMtDNA^Δ^ is located 1,752 bp upstream of a second EcoRI site in mtDNA^WT^, and 66 bp upstream of an EcoRI site in mtDNA^Δ^ (Fig. 3a). After three rinses in wash A at RT for 5 min, we hybridized 185 nM of both padlock probes under the published conditions^42^ at 37 °C for 40 min. To remove unbound padlock probes we rinsed sample in wash B (2 × SSC, 0.02% Triton X-100) at 37 °C for 5 min, and then in wash A three times each for 5 min at RT. Padlock probes were circularized using 0.1 U μl^−1^ T4 DNA ligase (NEB) in the supplied buffer supplemented with 500 μM of ATP (NEB) at 4 °C overnight. Samples were then rinsed in wash B at 37 °C for 5 min and three times in wash A at RT for 5 min. We performed the RCA reactions using 1 U μl^−1^ of Φ29 DNA polymerase (NEB) in the supplied buffer supplemented with 5% glycerol and 200 μg μl^−1^ of BSA at 37 °C for 40 min. Samples were rinsed in wash A three times at RT for 5 min, and then post-fixed in 4% formaldehyde in PBS with 0.3% Triton X-100 for 20 min each. After three rinses in wash A, we hybridized 250 nM of fluorophore-tagged detection probes (Supplementary Table 6) under the same conditions as for the padlock probe hybridization, at 37 °C for 90 min. The samples were rinsed three times in wash A at RT for 5 min before mounting them in VectaShield (Vector Laboratories) on a microscope slide.

The samples used for immunostaining were blocked in 5% BSA in wash A at RT for 1 h. After blocking, samples were rinsed three times in wash A at RT for 5 min, and stained with primary and secondary antibodies overnight at 4 °C. After primary and secondary staining, samples were extensively rinsed, four times in wash A at RT for 20 min each. Mitochondria were immunostained using mouse monoclonal (15H4C4) anti-ATP5A (Abcam #14748) at a 1/300 dilution and goat anti-mouse Alexa Flour 405 (Abcam #175661) at a 1/250 dilution.

### Imaging of tpRCA

Images of *in situ* tpRCA were acquired with the Olympus FV-1000 confocal microscope using a × 60 (numerical aperture=1.30) objective and 1,024 × 1,024 resolution at 12 bits per pixel. Each channel was scanned consecutively at 4 μs per pixel applying a threefold zoom. For each genotype, tissue from six flies was examined, with four or more images per fly, and a single 400 × 400 image is displayed in Figs 5b and 6b.

### Image quantification

ImageJ 2.0 was used for image analysis and quantification. For each colour channel, a threshold was determined from a channel's histogram and applied to remove background pixels. To quantify numbers of fluorescent loci (mCherry-Atg8a, Alexa Flour 488 and TAMRA), colour channels were split, converted to 8 Bit grey scale and then to binary images. Numbers of fluorescent foci were counted with a particle analysis tool in ImageJ. Total numbers of fluorescent foci counted were similar for all genotypes and samples.

### Quantification of mtDNA^Δ^ using tpRCA

In order for a padlock probe to be able to hybridize to its target, and to be used as a substrate for rolling-circle amplification, several things must happen. First, the 5′–3′ exonuclease must degrade one strand of mtDNA, beginning at the cleaved EcoRI site, to provide a probe hybridization site. Second, following hybridization of the padlock probe, Φ29 DNA polymerase must shorten the unprimed 3′-end so that it becomes located sufficiently near the probe hybridization site to function as a primer. Both of these exonucleolytic events are likely to be inhibited in a distance-dependent manner in an *in situ* preparation of formaldehyde fixed tissue, since *in vivo* mtDNA is bound to varying degrees, depending on context, by mtDNA-packaging proteins such as TFAM^1^. Larsson *et al.*^42^ observed such distance-dependent behaviour, with padlock probes located close to the restriction enzyme-created end being detected twice as frequently as those located 134 bp away. In our experiments, the mtDNA^Δ^ padlock probe is located 1.7 kb away from the exposed 3′-end in wild-type mtDNA, but only 66 bp away in mtDNA^Δ^. Thus, failure of one or both of the above exonucleases to process the intervening mtDNA provides a mechanistic basis for our ability to specifically detect mtDNA^Δ^. With respect to our ability to quantify the fraction of mtDNA^total^ represented by mtDNA^Δ^ using tpRCA, we note that target sites for mtDNA^Δ^ and mtDNA^total^ padlock probes are located at different distances from their closest EcoRI cut sites: 66 and 44 bases, respectively. Assuming that the ability of a 3′-end to prime tpRCA is a linear function of distance (based on the above proposed mechanisms, and the observations of Larsson *et al.*)^42^, we applied a correction factor for different efficiencies of RCA of the circularized padlock probes by adjusting the percentage of mtDNA^Δ^ (red) to mtDNA^total^ (green) loci on tpRCA images by 3/2.

### *mitoAflIII* fly feeding experiments

The P{*pIFM-mitoAflIII, pIFM-mitoT4lig, pIFM-*Gal4I}attP1/CyO male flies were used for feeding experiments. All flies were kept under the same conditions at 25 °C with a 12H/12H light and dark cycle. Twenty flies were introduced into a vial, and flies were transferred into a new vial every other day. Rapamicyn (99+%, Alfa Aesar) was dissolved in 100% ethanol to a 40 mM stock concentration. It was gradually diluted in water to a 200 μM working concentration. Drosophila food was prepared from Instant formula 4–24 (Carolina) dissolved in water with 200 μM rapamycin. Diluted ethanol alone was added to the control food. Newly enclosed flies were kept for 10 days on 200 μM of rapamycin.

### Quantification of mtDNA^Δ^ from *mitoAflIII* flies fed on supplemented diets

Total DNA was extracted from dissected IFMs of 10-day-old male flies. The same qPCR primers (Supplementary Table 4) and ΔΔCt quantification algorithm were used to estimate changes in levels of mtDNA^total^ and mtDNA^Δ^ molecules between the control flies and flies fed on food supplemented with rapamycin. Because each group (experimental and control) was fed on Instant formula 4–24 (Carolina), we could not normalize the fold change in mtDNA^Δ^ induced by rapamycin to the level of mtDNA^Δ^ in the *mitoAflII* flies fed on the standard fly food. Instead, changes in abundance of the mtDNA^total^ and mtDNA^Δ^ are presented as percentages relative to the respective estimates in the control flies, which were given the value of 100%. Four biological replicates were analysed for each feeding experiment.

### Statistical analysis

Statistical analysis was performed in JMP 8.0.2 by SAS Institute Inc. Observations from each genetic background were compared with corresponding values from *mitoAflIII* flies (Figs 5a and 6a). *P* values were calculated for a two-sample Student's *t*-test with unequal variance.

### Data availability

The sequence of plasmid used to generate *mitoAflIII* flies is deposited in GenBank under accession code KX696451.

## Additional information

**How to cite this article**: Kandul, N. P. *et al.* Selective removal of deletion-bearing mitochondrial DNA in heteroplasmic Drosophila. *Nat. Commun.* **7**, 13100 doi: 10.1038/ncomms13100 (2016).

## Supplementary Material

Supplementary Information Supplementary Figures 1-5, Supplementary Tables 1-6, Supplementary Methods and Supplementary References
