# Supplementary material for: Selective removal of deletion-bearing mitochondrial DNA in heteroplasmic Drosophila
Source: Nat Commun. 2016 Nov 14;7:13100. doi: 10.1038/ncomms13100 (PMC5114534; doi:10.1038/ncomms13100)
Supplement: Supplementary Information — Supplementary Figures 1-5, Supplementary Tables 1-6, Supplementary Methods and Supplementary References [file ncomms13100-s1.pdf]

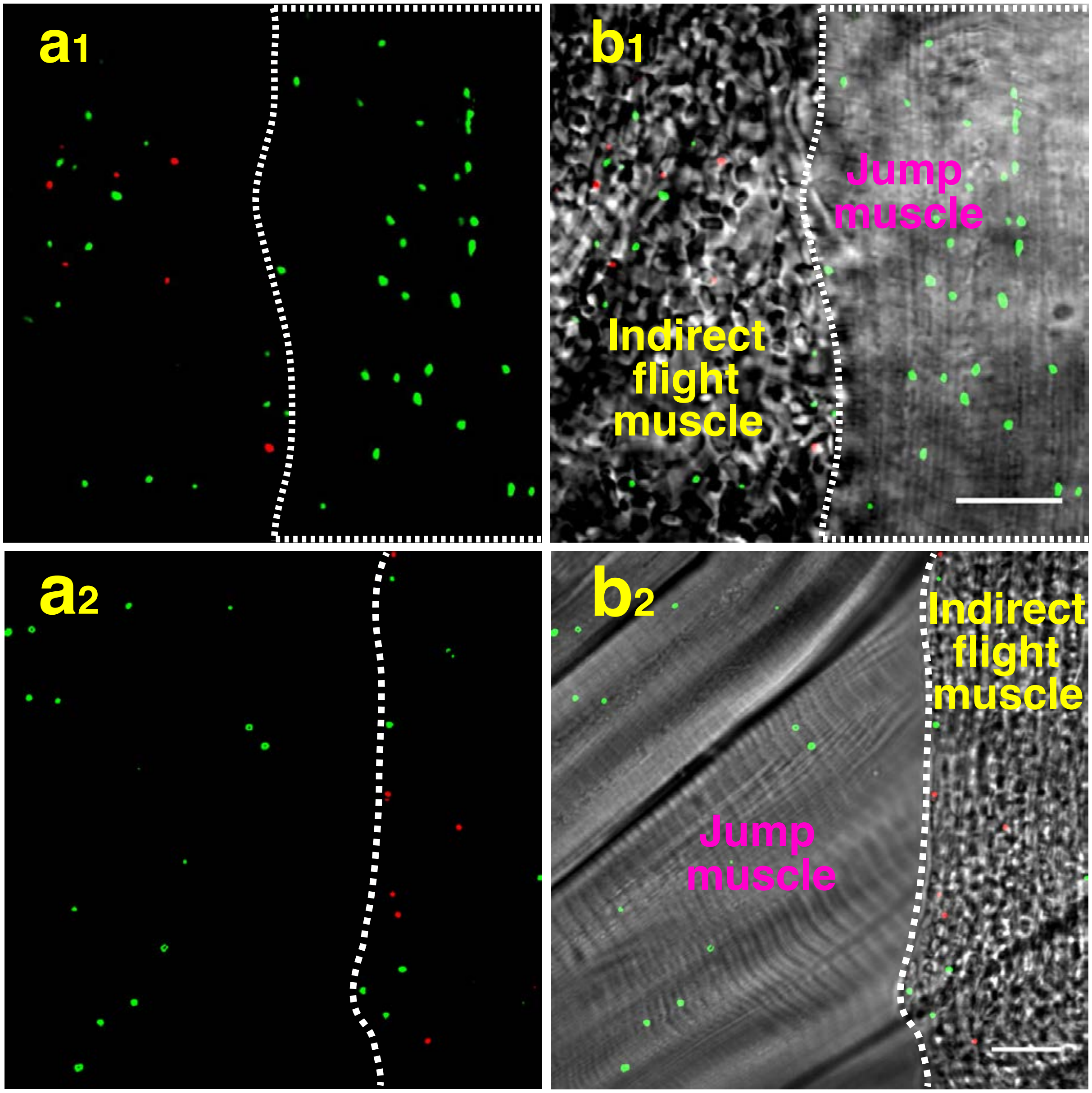

Supplementary Figure 1. tpRCA detects mtDNA$^{\Delta}$ in indirect flight muscle (IFM) of *mitoAflIII* flies. a$_{1\text{-}2}$, Red and green dots are observed only in the IFMs, in which *mitoAflIII* and *mitoT4 DNA ligase* are expressed, but not in the neighboring jump muscle, **b$_{1\text{-}2}$**. The IFMs and jump muscle have very different structures, and can be easily differentiated at the level of light microscopy. Scale bar is 10 μm (**b**).

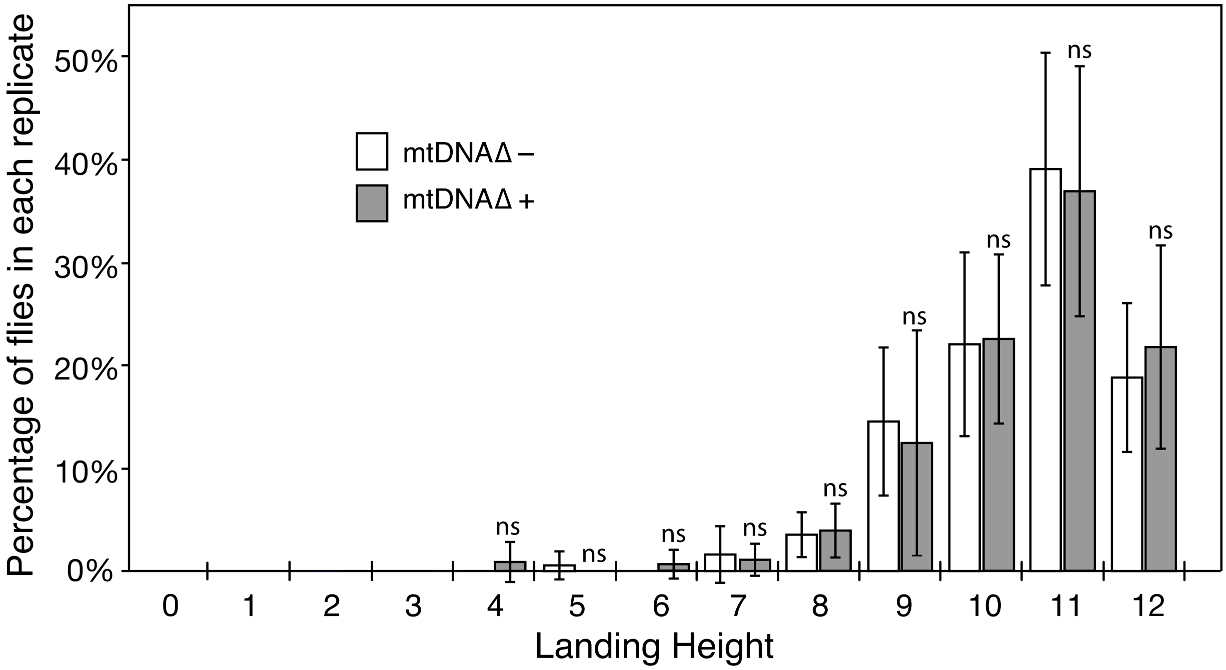

Supplementary Figure 2. *mitoAflIII* flies display no defect in flight performance. A flight assay[1-5] was used to compare flight performance between *mitoAflIII* (grey bars) and wildtype (white bars) ten-day-old male flies. Five groups of 30–50 flies of each type were introduced into the top of a 500-mL graduated cylinder whose internal walls were coated with paraffin oil. Wildtype flies quickly initiate horizontal flight, striking the wall close to the entry level, whereas poor fliers land at lower levels or at the bottom of the cylinder. Statistical significance was calculated with respect to percentage of total flies of each type that landed at each height using Student's *t*-test with unequal variance. Bars depict mean ± one standard deviation. P>0.05(ns).

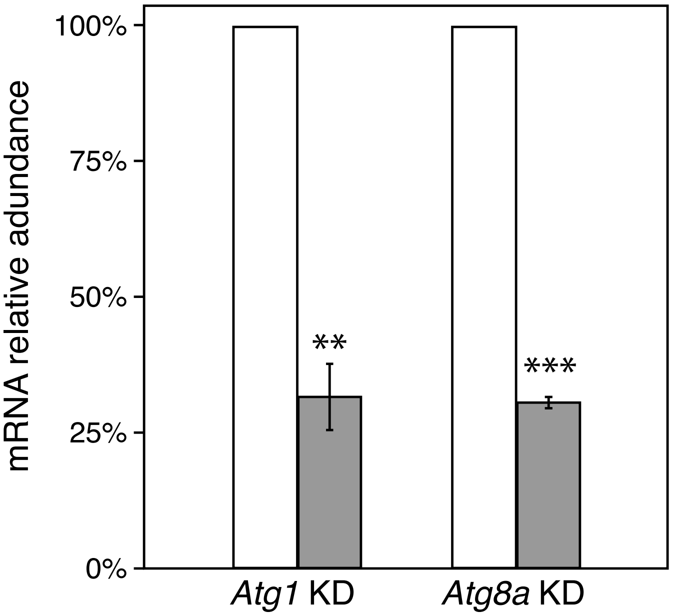

Supplementary Figure 3. *Atg1* and *Atg8A* RNAi (KD) using *pIFM-Gal4*. Relative percentages of *Atg1* and *Atg8a* mRNAs in the *pIFM-Gal4 >>pUASt-RNAi* flies (grey) are normalized to those in the *pIFM-Gal4* flies (white). Bars indicate mean ± one standard deviations. Statistical significance was calculated using Student's *t*-test with unequal variance. $P<0.01$** and $P<0.0001$***.

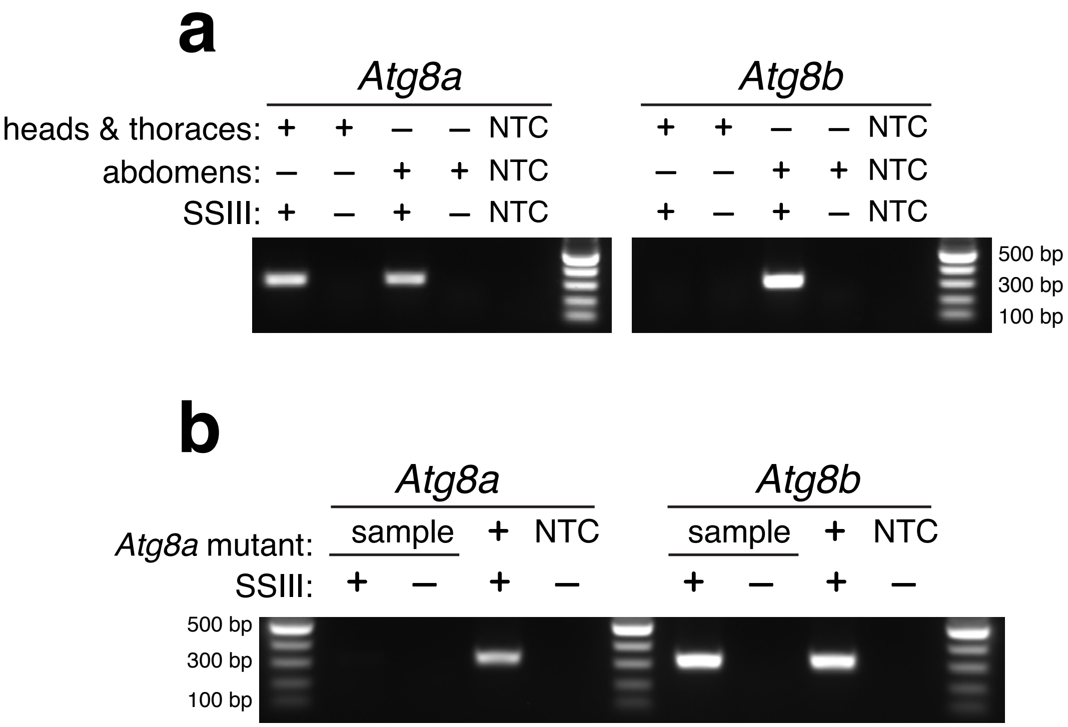

Supplementary Figure 4. *Drosophila Atg8a* and *Atg8b* transcript analysis in *Atg8a* mutant flies. *Drosophila* has two paralogs of yeast *Atg8.* **a**, Reverse transcription (RT) PCR analysis shows that *Atg8a* is expressed throughout male flies (heads, thoraces, and abdomens), whereas *Atg8b* transcripts are found only in male abdomens, reflecting expression in the testis (FlyBase.org)[6]. **b**, A P element insertion in the coding region of *Atg8a* (P{SUPor-P}Atg8a^{KG07569}, referred here as *Atg8a* mutant) causes a massive reduction in the level of *Atg8a* mRNA in the entire fly body. The mRNA abundance of *Atg8b* is not affected in *Atg8a* mutant males.

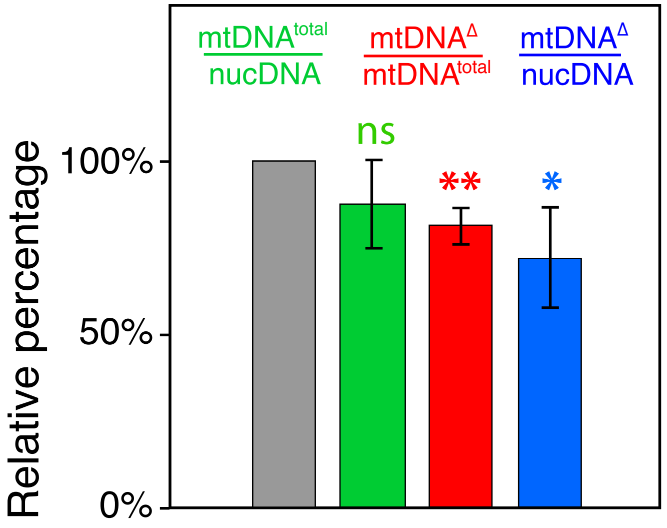

Supplementary Figure 5. Rapamycin feeding results in decreased mtDNA$^{\Delta}$ abundance in *mitoAflIII* flies. Relative abundances of total mtDNA (green) and mtDNA$^{\Delta}$ (red and blue) in flies fed on food supplemented with 200μM rapamycin were estimated using qPCR and normalized to those present in control *mitoAflIII* flies (grey). Colored bars indicate mean ± one standard deviations. Statistical significance is estimated as compared with the corresponding estimate in control flies using Student's *t*-test with unequal variance. P>0.05(ns), P<0.05*, and P<0.01**.

Supplementary Table 1. Insertions and their frequencies at the post-cleavage AflIII ligation site in mtDNA$^{\Delta}$

5'-GTATTTACTTA	2580bp	CATGTTTTTGTTAAA-3'
3'-CATAAATGAATGTAC	2580bp	AAAAACAATTT-5'

sequence at the ligated post-cleavage site	Insertion size (bp)	frequency
5'-GTATTTACTTAcatgCATGTTTTTGTTAAA-3'	4	6/20
5'-GTATTTACTTAcat CATGTTTTTGTTAAA-3'	3	6/20
5'-GTATTTACTTAcatg ATGTTTTTGTTAAA-3'	3	5/20
5'-GTATTTACTTAc CATGTTTTTGTTAAA-3'	1	2/20
5'-GTATTTACTTA CATGTTTTTGTTAAA-3'	0	1/20

Two primers flanking both AflIII sites amplify a fragment that spans mtDNA$^{\Delta}$ deletion from *mitoAflIII* flies. Because direct sequencing of PCR products resulted in an ambiguity at the ligated post-cleavage AflIII site, the fragment was cloned, and 20 clones were sequenced. The sequence at the top depicts double stranded sequences generated after cleavage at both AflIII sites, with the AflIII site indicated in blue. Sequences isolated through PCR and cloning, along with their frequency are indicated. Bases inserted at the ligation sites are shown in red.

Supplementary Table 2. Viability of progeny from cross between *pUASt-mitoT4lig* lines and Gal4 drivers

Gal4 driver	P{*pUASt-mitoT4lig*}attP1	P{*pUASt-mitoT4lig,pUASt-mitoAflIII*}attP1
da-Gal4	viable	lethal
elav-Gal4	viable	lethal
mef2-Gal4	viable	lethal
ey-Gal4	viable	lethal
r4-Gal4	viable	lethal

Expression of *mitoT4lig* driven by five Gal4 drivers did not cause observable defects in *Drosophila*. In contrast, co-expression with *mitoAflIII* resulted in lethality early in development.

Supplementary Table 3. Amounts of total mtDNA and mtDNA$^{\Delta}$ in IFMs estimated with qPCR and tpRCA

	qPCR, mtDNA^{all}/*3R:tub*				qPCR, mtDNA$^{\Delta}$/mtDNA^{total}			qPCR, mtDNA$^{\Delta}$/*3R:tub*			tpRCA, mtDNA$^{\Delta}$/mtDNA^{total}			
Genetic background	Sample number	Mean %	Std Dev	Std Err Mean	Mean %	Std Dev	Std Err Mean	Mean %	Std Dev	Std Err Mean	Sample number	Mean %	Std Dev	Std Err Mean
mitoAflIII	6	94.18	5.33	2.18	75.63	1.19	0.38	77.62	0.87	0.35	22	72.32	10.93	2.33
Atg1 KD	4	90.41	3.13	1.57	82.05	2.18	1.09	83.20	1.75	0.87	23	93.48	17.76	3.70
Atg8a KD	4	84.27	12.40	6.20	89.41	0.71	0.35	89.74	2.00	1.00	23	90.15	18.05	3.77
Atg8a mutant	5	91.64	6.47	2.89	89.40	2.17	0.97	91.31	3.23	1.44	21	95.95	15.42	3.37
Atg1(6B) OE	4	94.40	1.04	0.52	3.82	0.79	0.39	4.29	0.54	0.27	22	2.66	1.17	0.25
Atg8a OE	4	90.41	3.40	1.70	66.92	3.08	1.54	66.91	3.48	1.74	26	70.12	15.04	2.95
Parkin OE	4	76.48	4.73	2.37	5.08	0.32	0.16	4.26	0.32	0.16	22	2.47	1.14	0.24
PINK1 OE	4	91.34	5.51	2.75	26.24	2.67	1.33	27.49	0.70	0.35	24	16.19	4.91	1.00
Parkin OE in *Atg8a* mutant	4	92.25	2.32	1.16	82.26	1.93	0.97	84.19	1.17	0.59	26	67.20	20.98	4.11
Mfn KD	4	94.51	0.28	0.14	39.16	1.29	0.65	43.29	3.32	1.66	26	26.05	8.99	1.76
Drp1 OE	4	94.57	0.76	0.38	88.13	1.89	0.94	90.32	1.82	0.91	28	90.85	17.86	3.37
ATPIF1 OE	4	92.38	3.15	2.18	68.28	8.53	4.26	69.19	8.40	4.20	N/A	N/A	N/A	N/A
ATPIF1 OE in Mfn KD	4	89.69	5.32	2.65	11.61	0.71	0.35	11.43	0.98	0.49	N/A	N/A	N/A	N/A

All genetic backgrounds contained P{*pIFM-mitoAflIII, pIFM-mitoT4lig, pIFM-Gal4*}attP1 transgene (*mitoAflII*), in an otherwise wildtype background (top row), or in combination with other mutations or transgenes supporting overexpression (OE) or knock down (KD) of a tested gene. To compare relative estimations calculated with qPCR and tpRCA on the same scale, we computed normalized percentages relative to *mitoAflIII* flies. A fold difference was calculated between relative amounts in *mitoAflIII* alone and in combination with a tested gene backgrounds using qPCR (see methods), then the fold difference was normalized to the corresponding percentage estimation in *mitoAflIII* flies (Fig. 2d). P-element insertion in a coding area of *Atg8a* (*Atg8a* mutant) caused a massive reduction in *Agt8a* expression and is thought to represent a strong hypomorph or null mutant (Supplementary Fig. 4)[7]. tpRCA was used to visualize and quantify mtDNA$^{\Delta}$ and mtDNA^{total} molecules in whole mounts of *Drosophila* IFMs. Amounts of mtDNA$^{\Delta}$ were estimated directly from confocal images as percentage of total mtDNA (mtDNA^{total}), multiplied by 3/2 (see methods for details).

Supplementary Table 4. Primers for quantification of mtDNA$^{\Delta}$ amount with qPCR

Name	Estimate	5′- Sequence- 3′	Amplicon size (bp)
mtAflIII$^{\Delta}$	mtDNA$^{\Delta}$	5′-CATATTTGTCGAGACGTTAATTATGGTTG-3′<br>5′-GAATTCGGCAAAAATAATATTCGCCTG-3′	129
mt:CytB	mtDNA^{WT}	5′-TAGTGTTAATCATATTTGTCGAGACGTT-3′<br>5′-ATATGAACCGTAATAAATTCCTCGTCC-3′	130
mt:ND5	mtDNA^{total}	5′-GAAGTAAAGCTACATCCCCAATTCG-3′<br>5′-GGTGAGATGGTTTAGGACTTGTTTC-3′	113
3R:tub	nuclear DNA (nucDNA)	5′-TATAAGTAAAGGCAGCAGGGAGAC-3′<br>5′-ATCTGGGTACTCTTCCTCTCCATC-3′	121

Supplementary Table 5. Primers for reverse transcription (RT) qPCR

Annotation number	Gene symbol	5′- Sequence- 3′	Amplicon size (bp)
CG10967	*Atg1*	5′-CAGGAGGACGAGAACACGGTGTC-3′	155
		5′-GGAAGGTTCTTTGGCACCAGCAC-3′	
CG32672	*Atg8a*	5′-TATCCAGACCGTGTGCCCGTC-3′	136
		5′-GTGGATGCGCTTGCGAATGAGG-3′	
CG2135	*βGlu*	5′-TGGAGGGCATGCACTCACTTC-3′	147
		5′-GGTCCGAAAATCGGCGAAGTTCC-3′	
mitoAflIII	*AflIII*	5′-AGCTGTAGATCTAAAAGGCGGCCG-3′	141
		5′-ATAGCAGCTGGCGTGGAATTGGC-3′	

Supplementary Table 6. Padlock probes and corresponding detection probes

Probe	Estimate	5′- Sequence- 3′
ppMtDNA^{total}	mtDNA^{total}	5'-P-CTTCAATATATTCATAAGCTAGTCTTGTTACGT TTAAACCTCAACGTACTTGATTGGCTCCTTCTTAGTCA GTCTGCAATAGTAAATGGAG-3′
dpMtDNA^{total}–Alexa®488	tpRCA product of ppMtDNA^{total}	5'-Alexa488-CCTCAACGTACTTGATTGGCTCC-3'
ppMtDNA$^{\Delta}$	mtDNA$^{\Delta}$	5'-P-GTAATAATCAACCATAATTATCTTTCTTCGTCC TCATTGCTGCTGCTGTACTACTAGTTCTTGTACTTACT CACCGTTAGCATGTAAAGTTC-3′
dpMtDNA$^{\Delta}$–TAMRA	RCA product of ppMtDNA$^{\Delta}$	5′-TAMRA-CCTCATTGCTGCTGCTGTACTAC-3'

The probes were purchased from Integrated DNA Technologies®. P, 5' phosphate. Target-complementary sequences of padlock probes are underscored and tag sequence segments of padlock probes used for fluorescence detection are color coded.

Supplementary Methods

qPCR quantifiction of relative amounts of mtDNA$^{\Delta}$. To quantify the amounts of mtDNA$^{\Delta}$ relative to both mtDNA^{total} and nuclear DNA (nucDNA) we applied the ΔΔCt algorithm as follows:

Normalizer #1 (mtDNA^{total}, *mt:NADH5*)

$\Delta Ct_{mtDNA^{\Delta} sample} = Ct_{mtDNA^{\Delta}} - Ct_{mtDNA^{total}}$

$\Delta Ct_{mtDNA^{\Delta} reference} = Ct_{mtDNA^{\Delta}} - Ct_{mtDNA^{total}}$

$\Delta\Delta Ct = \Delta Ct_{mtDNA^{\Delta} sample} - \Delta Ct_{mtDNA^{\Delta} reference}$

Fold difference = $2^{-\Delta\Delta Ct}$

Normalizer #2 (nucDNA, *3R:Tube*)

$\Delta Ct_{mtDNA^{\Delta} sample} = Ct_{mtDNA^{\Delta}} - Ct_{nucDNA}$

$\Delta Ct_{mtDNA^{\Delta} reference} = Ct_{mtDNA\Delta} - Ct_{nucDNA}$

$\Delta\Delta Ct = \Delta Ct_{mtDNA^{\Delta} sample} - \Delta Ct_{mtDNA^{\Delta} reference}$

Fold difference = $2^{-\Delta\Delta Ct}$

RT-qPCR quantifiction of transcript abundance. We calculated a fold difference in transcript number of a gene of interest (GOI) following Pfaffl[8] to account for differences in amplification efficiencies of used primers using the following equation:

$\Delta Ct_{GOI} = Ct_{GOI}^{test} - Ct_{GOI}^{reference}$

$\Delta Ct_{\beta Glu} = Ct_{\beta Glu}^{test} - Ct_{\beta Glu}^{reference}$

E = efficiency from a standard curve, E= $10^{-1/slope}$

Fold difference = $E_{GOI}^{\Delta Ct(GOI)} / E_{Mlc2}^{\Delta Ct(\beta Glu)}$

Supplementary References:

1 Benzer, S. Genetic dissection of behaviour. *Sci. Am.* **229**, 24-37 (1973).

2 Banerjee, S., Lee, J., Venkatesh, K., Wu, C. F. & Hasan, G. Loss of flight and associated neuronal rhythmicity in inositol 1,4,5-trisphosphate receptor mutants of Drosophila. *J. Neurosci.* **24**, 7869-7878, doi:10.1523/JNEUROSCI.0656-04.2004 (2004).

3 Walker, D. W. & Benzer, S. Mitochondrial “swirls” induced by oxygen stress and in the Drosophila mutant hyperswirl. *Proc. Natl. Acad. Sci. USA* **101**, 10290-10295, doi:10.1073/pnas.0403767101 (2004).

4 Venkiteswaran, G. & Hasan, G. Intracellular Ca2+ signaling and store-operated Ca2+ entry are required in Drosophila neurons for flight. *Proc. Natl. Acad. Sci. USA* **106**, 10326-10331, doi:10.1073/pnas.0902982106 (2009).

5 Sadaf, S., Birman, S. & Hasan, G. Synaptic activity in serotonergic neurons is required for air-puff stimulated flight in Drosophila melanogaster. *PLoS One* **7**, e46405, doi:10.1371/journal.pone.0046405 (2012).

6 The modENCODE Consortium. Identification of Functional Elements and Regulatory Circuits by Drosophila modENCODE. *Science* **330**, 1787-1797, doi:10.1126/science.1198374 (2010).

7 Nezis, I. P. *et al.* Ref(2)P, the *Drosophila melanogaster* homologue of mammalian p62, is required for the formation of protein aggregates in adult brain. *J. Cell Biol.* **180**, 1065-1071, doi:10.1083/jcb.200711108 (2008).

8 Pfaffl, M. W. in *A-Z of Quantitative PCR* (ed Stephen A. Bustin) 87-120 (International University Line, 2004).
